# SECOND CLASS CARE: PATTERNS OF LGBTQ+ PATIENT EXPERIENCES AND RECEIPT OF PREVENTIVE CARE IN MIDLIFE AND OLDER AGE

**DOI:** 10.1093/geroni/igae098.1203

**Published:** 2024-12-31

**Authors:** Nathaniel Tran, Tara McKay, Gilbert Gonzales, Carrie Fry, Stacie Dusetzina

**Affiliations:** University of Illinois Chicago, Chicago, Illinois, United States; Vanderbilt University, Nashville, Tennessee, United States; Vanderbilt University, Nashville, Tennessee, United States; Vanderbilt University Medical Center, Nashville, Tennessee, United States; Vanderbilt University Medical Center, Nashville, Tennessee, United States

## Abstract

The objectives of this study were to (1) Identify latent classes of LGBTQ+ patient experiences using seven indicators of clinical and cultural competence, (2) Identify sociodemographic characteristics associated with class membership, and (3) Estimate the association between class of patient experiences and receipt of preventive care. The sample included 954 LGBTQ+ adults aged 50-76 from the Vanderbilt University Social Networks, Aging, and Policy Study. Respondents reported on seven indicators of their clinician’s LGBTQ+ clinical and cultural competency. Latent class analysis was conducted to identify patterns of LGBTQ+ patient experiences. We estimated marginal effects of class membership on preventive care using logistic regression. Three latent classes emerged: 34% assigned to the Affirming class, 60% to the Neutral class, and 6% to the Discriminatory class. Gender identity, sexual orientation, race/ethnicity, state of residency, and HIV status significantly predicted class membership (all p< 0.01), but not education, income, health insurance, or chronic conditions. Compared to those the Affirming class, individuals in the Neutral class were 12.4pp less likely to have ever been tested for HIV and 17.1pp less likely to have been recently tested for HIV, both p< 0.001; those in the Discriminatory class were 12.2pp less likely to have recently received an influenza vaccination and 14.8pp less likely to have recently completed a colorectal cancer screening, both p< 0.05. In the absence of explicitly affirming healthcare experiences, LGBTQ+ patients are less likely to receive timely preventive services. Increasing the capacity of health systems to provide LGBTQ+ affirming healthcare may advance health equity.

